# Supplementary material for: Text speaks louder: Insights into personality from natural language processing
Source: PLoS One. 2025 Jun 18;20(6):e0323096. doi: 10.1371/journal.pone.0323096 (PMC12176201; doi:10.1371/journal.pone.0323096)
Supplement: S1 File — (DOCX) [file pone.0323096.s001.docx]

# Tables

## Table 1

Accuracies of the prediction of personality using Essays dataset

| **Paper** | **Method used** | **Measure** | **O** | **C** | **E** | **A** | **N** |
| --- | --- | --- | --- | --- | --- | --- | --- |
| [(Mehta Fatehi et al., 2020)](https://www.zotero.org/google-docs/?qxRBDh) | BERT-base + MLP | Accuracy | 0.646 | 0.592 | 0.6 | 0.588 | 0.605 |
| [(Majumder et al., 2017)](https://www.zotero.org/google-docs/?FkfmjA) | CNN + Mairesse sMLP/MP | Accuracy | 0.611 | 0.567 | 0.580 | 0.567 | 0.573 |
| [(Mohades Deilami et al., 2022)](https://www.zotero.org/google-docs/?Sfnpu5) | CNN + AdaBoost-2channel | Accuracy | 0.605 | 0.649 | 0.618 | 0.599 | 0.620 |
| [(El-Demerdash et al., 2022)](https://www.zotero.org/google-docs/?RSjdF3) | BERT + MLP | Accuracy | 0.643 | 0.589 | 0.599 | 0.588 | 0.601 |
| [(Xue et al., 2021)](https://www.zotero.org/google-docs/?Z1cDTX) | SEPRNN | Accuracy | 0.631 | 0.574 | 0.589 | 0.574 | 0.595 |
| [(Ramezani Feizi-Derakhshi & Balafar, 2022)](https://www.zotero.org/google-docs/?hu2l6O) | KGrAt-Net | Accuracy | 0.722 | 0.734 | 0.742 | 0.712 | 0.709 |
| (Yan et al., 2024) | DKSGAT | Macro F1 | 0.708 | 0.688 | 0.715 | 0.742 | 0.699 |
| (Sun et al., 2018) | 2CLSTM | Accuracy | 0.541 | 0.535 | 0.556 | 0.588 | 0.567 |
| [(Ramezani, Feizi-Derakhshi, Balafar, et al., 2022)](https://www.zotero.org/google-docs/?zfAA2L) | Ensemble modeling | Accuracy | 0.563 | 0.591 | 0.642 | 0.603 | 0.611 |

## Table 2

Accuracies of the prediction of personality using MBTI dataset

| **Paper** | **Method used** | **Measure** | **I/E** | **N/S** | **T/F** | **J/P** |
| --- | --- | --- | --- | --- | --- | --- |
| (Mehta et al., 2020) | BERT-large + MLP | Accuracy | 0.788 | 0.863 | 0.672 | 0.672 |
| (Yan et al., 2024) | DKSGAT | Macro F1 | 0701 | 0.668 | 0.804 | 0.695 |
| (Keh & Cheng, 2019) | BERT | Accuracy | 0.758 | 0.744 | 0.757 | 0.719 |
| (Bin Tareaf, 2022) | BERT | Accuracy | 0.540 | 0.534 | 0.540 | 0.545 |
| (Bin Tareaf, 2022) | XGBoost | Accuracy | 0.510 | 0.497 | 0.497 | 0.480 |
| (Bin Tareaf, 2022) | SVM | Accuracy | 0.472 | 0.517 | 0.506 | 0.517 |
| (Chen et al., 2023) | Random Forest | Accuracy | 0.777 | 0.860 | 0.702 | 0.596 |
| (Kerz et al., 2022) | BERT + PSYLING Ensemble | Accuracy | 0.854 | 0.922 | 0.857 | 0.825 |

## Table 3

Number of cases in the Essays dataset based on the personality labels

| **Personality trait** | **Number of positive cases** | **Number of negative cases** |
| --- | --- | --- |
| Openness to Experience | 1272 | 1196 |
| Conscientiousness | 1254 | 1214 |
| Extraversion | 1277 | 1191 |
| Agreeableness | 1310 | 1158 |
| Neuroticism | 1233 | 1235 |

## Table 4

Number of cases in the MBTI dataset based on the personality labels

| **MBTI letters** | **Frequencies** |
| --- | --- |
| I | 6676 |
| E | 1999 |
| N | 7478 |
| S | 1197 |
| F | 4694 |
| T | 3981 |
| J | 3434 |
| P | 5241 |

# Figures

## Figure 1


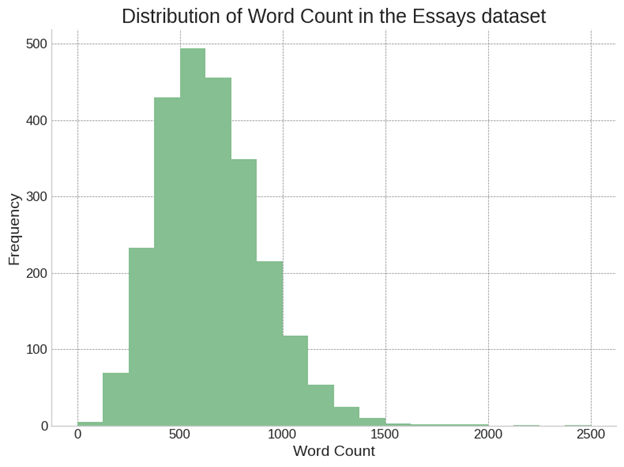


## Figure 2


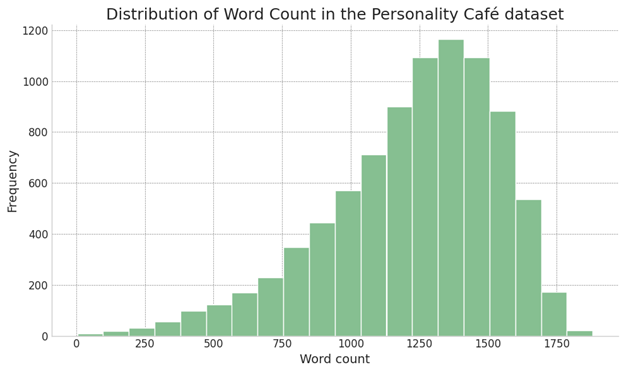


## Figure 3


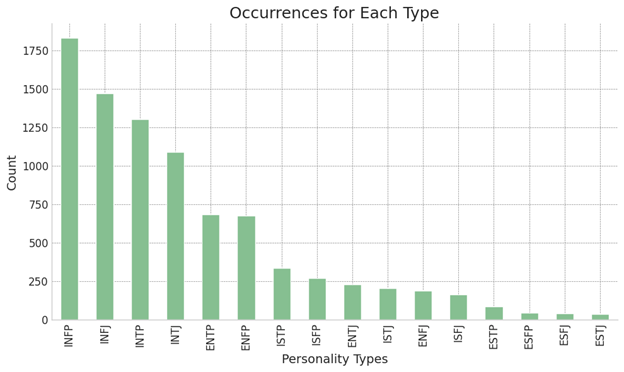


## Figure 4


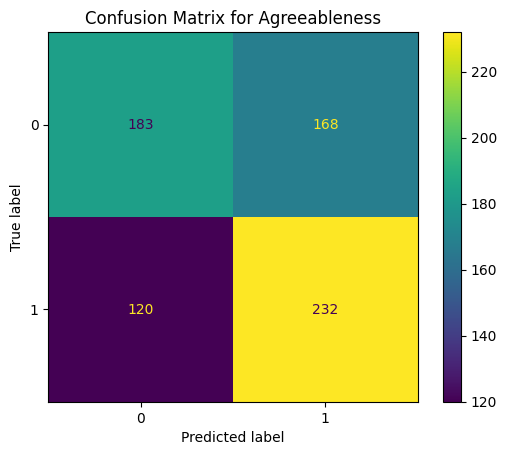


## Figure 5


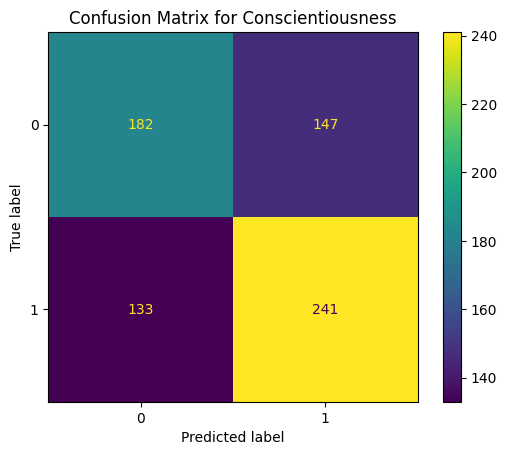


## Figure 6
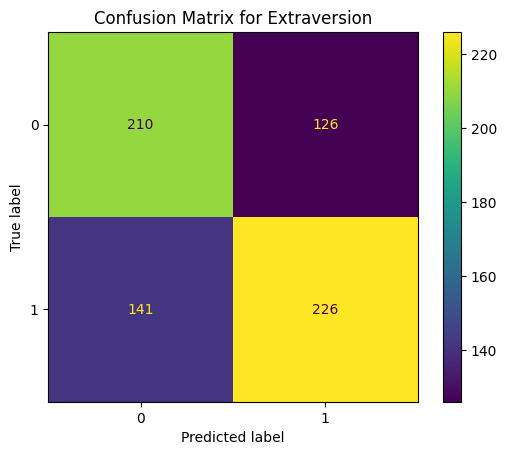


## Figure 7


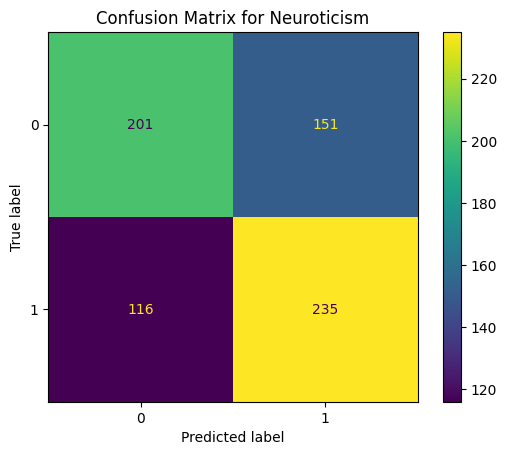


## Figure 8


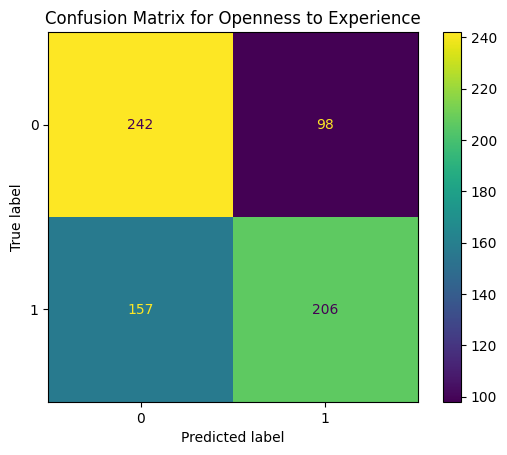


## Figure 9

##
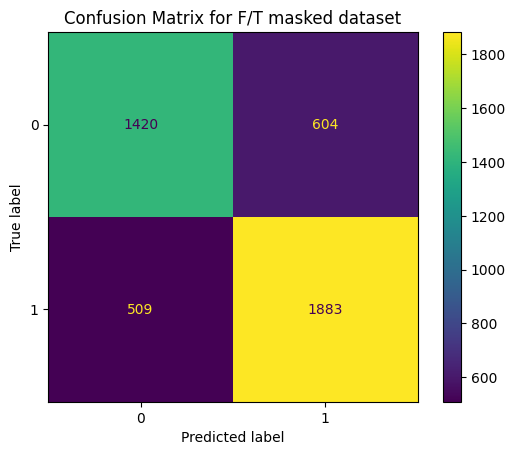


## Figure 10

##
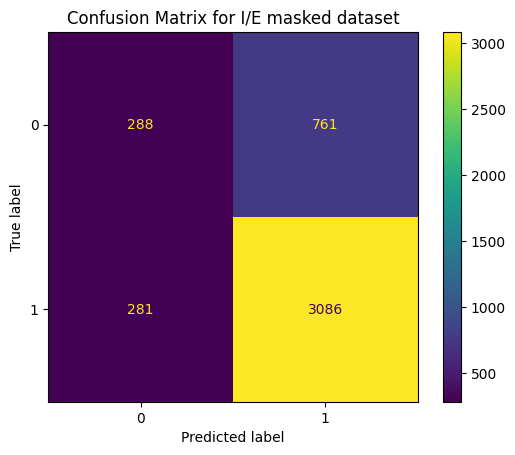


## Figure 11

##
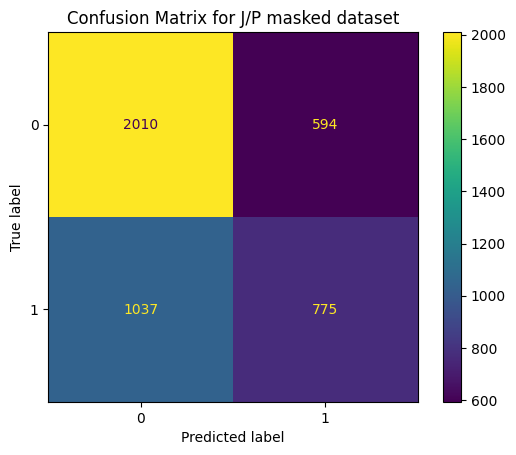


## Figure 12

##
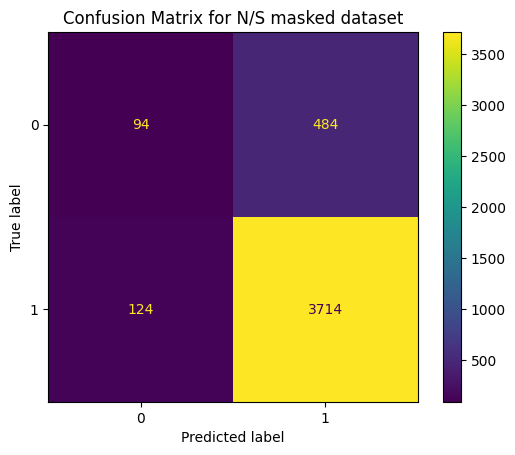


## Figure 13


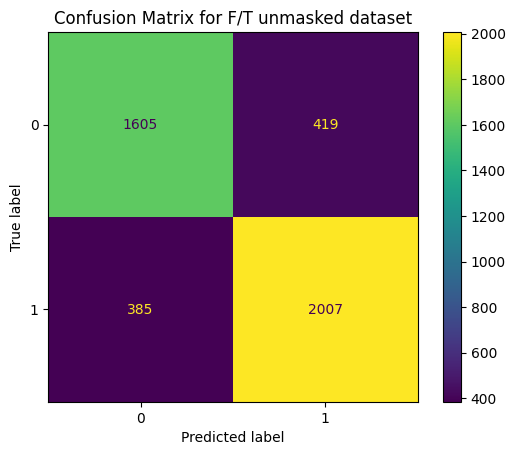


## Figure 14


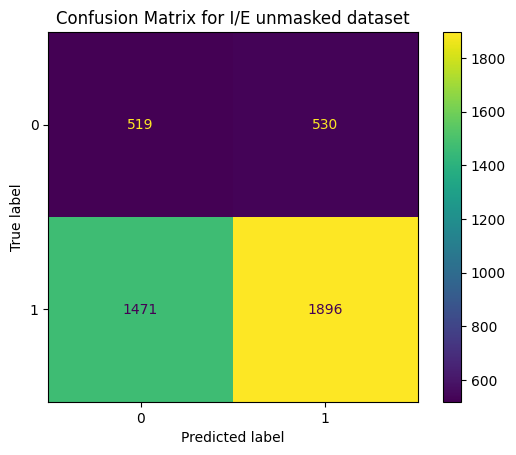


## Figure 15


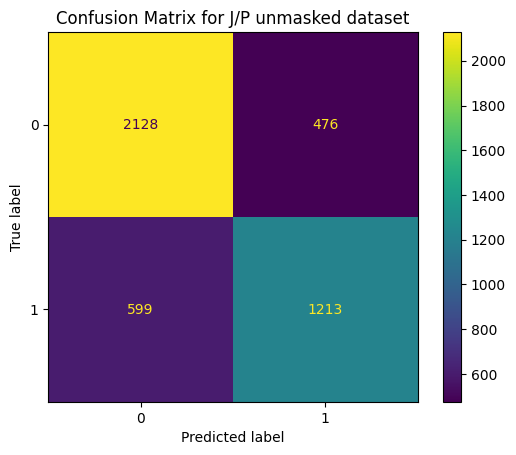


## Figure 16


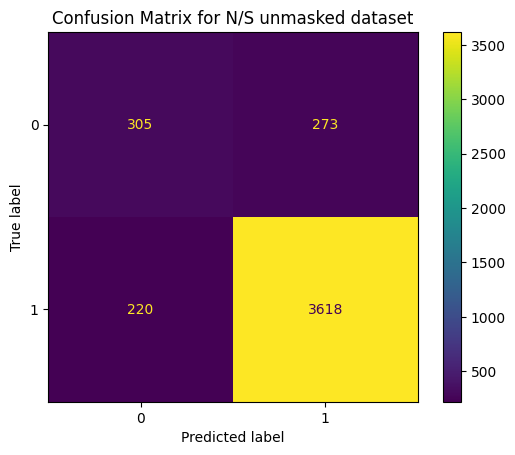


# Annex

## A1

Elements included in both the Informative Dirichlet Prior and for the fine-tuning

| Full Types (Uppercase) | Short Types (Lowercase) |
| --- | --- |
| ENFJ | enfj |
| ENFP | enfp |
| ENTJ | entj |
| ENTP | entp |
| ENFJs | enfjs |
| ENFPs | enfps |
| ENTJs | entjs |
| ENTPs | entps |
| ESFJ | esfj |
| ESFP | esfp |
| ESTJ | estj |
| ESTP | estp |
| ESFJs | esfjs |
| ESFPs | esfps |
| ESTJs | estjs |
| ESTPs | estps |
| INFJ | infj |
| INFP | infp |
| INTJ | intj |
| INTP | intp |
| INFJs | infjs |
| INFPs | infps |
| INTJs | intjs |
| INTPs | intps |
| ISFJ | isfj |
| ISFP | isfp |
| ISTJ | istj |
| ISTP | istp |
| ISFJs | isfjs |
| ISFPs | isfps |
| ISTJs | istjs |
| ISTPs | istps |

Other elements included only in the fine tuning:

E, N, S, F, T, J, P, Es, Ns, Ss, Fs, Ts, Js, Ps, EN, En, en, FJ, fj, FP, fp, TJ, tj, TP, tp, ENs, Ens, ens, FJs, fjs, FPs, fps, TJs, tjs, TPs, tps, NE, Ne, ne, NI, Ni, ni, SI, Si, si, TE, Te, te, FI, Fi, fi, NEs, Nes, nes, NIs, Nis, nis, SIs, Sis, sis, TEs, Tes, tes, FIs, Fis, fis, NF, Nf, nf, NT, Nt, nt, TJ, Tj, tj, SF, Sf, sf, ST, St, st, NFs, Nfs, nfs, NTs, Nts, nts, TJs, Tjs, tjs, SFs, Sfs, sfs, STs, Sts, sts, ENF, enf, ENT, ent, ESF, esf, EST, est, ENFs, enfs, ENTs, ents, ESFs, esfs, ESTs, ests, NFJ, nfj, NFP, nfp, NTJ, ntj, NTP, ntp, NFJs, nfjs, NFPs, nfps, NTJs, ntjs, NTPs, ntps, SFJ, sfj, SFP, sfp, STJ, stj, STP, stp, SFJs, sfjs, SFPs, sfps, STJs, stjs, STPs, stps, INF, inf, INT, int, ISF, isf, IST, ist, INFs, infs, INTs, ints, ISFs, isfs, ISTs, ists.
