# Supplementary material for: Text speaks louder: Insights into personality from natural language processing
Source: PLoS One. 2025 Jun 18;20(6):e0323096. doi: 10.1371/journal.pone.0323096 (PMC12176201; doi:10.1371/journal.pone.0323096)
Supplement: S2 File — (DOCX) [file pone.0323096.s002.docx]

## Traits

### Agreeableness

- General positive judgments about others (+)
  - “She’s just good luck, I guess, I’m so lucky to have supportive, caring parents, they have taught me that school comes first so it is not really that hard for me to be away from home…”.
  - “He’s seriously the best thing to happen to me for a really long time. [...] He is so sweet and he’s really in touch with his sensitive side, and that really turns me on in a guy. I like when guys cry too…”.
  - “Oh, well, I ended up pledging Alpha Chi Omega and I absolutely love it! The sweetest girls in the world are in there and they are so much fun! I have made many friendships that will last for a lifetime already…”.
- Is considerate and kind to almost everyone (+)
  - “I feel really bad about my dad having to pay so much money for me to get my education here. I want to help out more. I hate it when my parents have to put up money for me…”.
  - “I am very accepting of all types of personal tastes that others may have…”.
  - “Sometimes I wonder how the students at UT feel away from home. I know that my first semester here I was extremely homesick…”.
  - “My dad really works too hard so this will be a time for them to relax and have some fun.”
  - “I forgive my mom for how she feels because she just doesn't know any better…”.
- Cooperative with others (+)
  - “I 've been thinking about going on a mission for the church also…”.
- Start quarrels with others (-)
  - “I hope I can find some program to rid of these stupid pop up windows. They are so annoying. I want to find the people that create these and ring their necks…”.
- Criticize others (-)
  - “I look at all these people and I criticize them all the time. I even criticize scott and I wish I didn't because then I think life would be much easier to live in…”.
  - “Jessica Simpson is such a bitch, all through middle school me and my friends would have killed to get some * from her".
  - “I poked several holes in the Christian philosophy and in the Bible. He wasn't able to counter my arguments, and he responded only by saying that he has faith. This epitomizes human ignorance…”.
  - “I hate Stacy so much, but sometimes I just love too much. I wish I could sever all ties with her -- oh well shit happens. She just makes me so angry, but my anger is a sign that I haven't gotten rid of my ties to her…”.
- Finds fault in others (-)
  - “Right now I'm listening to some music that my parents would consider useless noise. It upsets me that they think that the music from their generation is so perfect and magical and music from my generation is crap. And they think that all of the musicians I listen to are miserable drug addicted singers…”.
  - “If the person blindly tries to refute the statement without the use of reason. They are a tool…”.
  - “John's a good guy but never helps out with calculus. Some friend […] The damn airwave guy was supposed to come down and take care of the internet problem but it turns out he doesn't give a rat's ass about the internet or my calculus or my stream of consciousness, man he sucks big time...”.
  - “Not like David does though. Jesus, talk about chemical imbalance. That fucker has some serious problems. Ever since the mushroom incident I haven't looked at him the same. Crazy guy. And his mom is such a bitch, and his alcoholic dad, and his pothead brother. With his mom dying I hope he's alright. Poor guy. It's a shame he doesn't care…”.
  - “Will is annoying me. He has this dumb ass book he will not put down. I hate competing for his attention. Asshole. Oh yeah, Will is my boyfriend…”.
- Don’t have a forgiving nature (-)
  - “This guy just bumped into me and did not even apologize. I hate it when people do that. He just walks by like it 's okay to knock people into a damn computer screen. Jerk…”.
  - “[...] wonderful because Richard was so horrible. I wonder what Richard is doing. I wonder if he is ended up going SAE. I wonder if he has a girlfriend. I wonder if he is a possessive weirdo to her…”.
  - “I was really insulted by those stupid papers that basically said that I was in the lowest percentile of students at this university…”.

### Conscientiousness

- Productivity (+)
  - "I am pretty proud of myself today, for i got much more accomplished than I thought I would. I figured that after my last class I would just take a nap. But instead, I went to the gym and had a great workout..."
  - “I have gotten all of my work for this week done already so I'm at ease about that…”.
- Planning (+)
  - “I want to meet new people as well as keep my old friends. I hope the meetings at fsa and vsa will aide me in meeting new people…”.
  - “It's always good to make a plan of everything that you want to do in a day. I always have to do lists. They are so useful, and I feel so organized when I make them. And when I finish everything on one list, I feel so good. It is a great feeling…”.
- Lazy, disorganized, careless (-)
  - “My interests change day-to-day. One day I want to do this, then the next day it's this other…”.
  - “I took French in high school and it was really tough. But maybe that was because I didn't really work hard my first two years of high school and I never really paid attention in class…”.
  - “I usually loose these pens and pencils within 3 or 4 weeks…”.
  - “The tv is kind of annoying i want to turn it off but it's so far away. College is making me lazy…”.
  - “im so glad i can type fast or this would be terribly frustrating. i don't really type correctly though.. but as long as its fast i don't really care…”.
  - “I have to do one of those for English and I haven't even thought about. I'm a procrastinator it will probably be the night before and I hadn't even started on it…”.
  - “i just need close people that all is there to be with and together nothing more the one, but any way I get mad at my self for not doing what I need to in school because I actually like it all I love learning but I just can’t put my ass in the chair and sit. Once I do I get it done and have fun doing it, I still feel weird just lackadaisical…”
  - “[...] I clinked on some link and this page transferred to somewhere else and everything was lost. That sucks. That sucks. That sucks. [...] the link I clicked was so stupid. It was in my friends profile. quotes from my calc teacher last year. They weren't funny when I read them. I knew they wouldn't be anyway. But I had to click on the damn link. Wish I could get that moment back. About one hundred quotes and not one was funny…”.
  - “I wish I had a better spelling sense I guess I could blame it all on my first grade teacher…”.
  - “Damn, I miss Georgie. stupid punk with too much time on his hands and he knows it. Procrastinator in the making just like me…”

### Extraversion

- Sociability (+)
  - “I just went to a x th anniversary party for my grandparents last night. I got up to speak with the other grandkids…”.
  - “I would hate it if it turned out to be similar to a sorority because I hated that kind of stuff, it's so fake and I have enough friends without having to pay for them…”.
  - “I would be a bad boy and want to mess around on her…”.
  - “The head ra (resident advisor) is always in our room hanging out and drinking our beer…”.
  - “I was always sweet to everyone but the girls always taked bad about me because I was friends with their boyfriends or was with the guy they wantred…”.
  - “[...] yet they give us all these mixers to go to to meet people which I love to do…”.
  - “My friends and I are all going to hardrock cafe that night to eat and then we might go to sixth street and party afterward…”.
  - “I wish I could be more disciplined like that. I would be much happier with myself. But, I am having so much fun. Will study later tonight at barnes and noble's with Amy. We'll see how that place is for studying…”.
  - “I always hear my friends going up, and saying "Aaron, how in the hell does that guy have that beautiful girl, when he is so damn ugly”.
  - “She couldn’t be any sweeter, but she is going home for the second weekend in a row. I can’t wait to take her to a frat party with me, she needs to let go and show her wild side, I’m really excited about that…”.
  - “I just came back to my dorm from hobby lobby. I went there with my friends Lauren and Rachel to get crafts and other supplies for our nothing books. Nothing books are like scrapbooks, and we have to make them for our sorority, alpha epsilon phi…”.
- Enthusiasm (+)
  - “I really like that class! it's pretty interesting, and the professor is pretty cool and not so boring…”.
  - “Dang, there are a lot of hot chicks at ut…”.
  - “Even tho she’s not in psych. its really interseting to her. But it is to me too!! I really like this class.. ok.. this sounds like total sucking up. But don't worry I hate sucking up. Im totally honest.. it gets me into trouble sometimes.. i don't know who im sposed to b typing to or if im not sposed to b talking to someone at all.. well its ok.. its stream of conciousness rite? rite!! I could go for some starbucks rite now ! !.. i love that place…”.
  - “but no. That's not me. There's just something in me that won't let me settle on”.
  - “It's pretty funny, one night the night guard knocked on our door because someone had made a noise complaint…”.
  - “I ended up pledging alpha chi omega and I absolutely love it! The sweetest girls in the owrld are in there and they are so much fun!...”.
- Energy (+)
  - “ahh!! I would like to have…”.
  - “my thoughts move faster than i write so its kind of hard because i have so many thoughts yet my hands can only go so fast…”.
  - “Damn man. I need to go back to the gym…”.
  - “I played basketball, ran track, softball, was a cheerleader all thourhg school, did student council, sisters of service, fellowship of christian athletes, and took a few leadership roles and it was great…”.
- Assertiveness (+)
  - “I hope I can fit them all in to my busy schedule…”.
  - “Dinner here at dobie really isn’t worth the amount that we pay to stay here. its decent.. but i expected better…”.
  - “I'm. finally. I could be in a management position at the pool I lifeguard at in the summers, next summer…”.
  - “I always argued with our sponsor because she didn't like me and I had no respect for her because she cheated on her husband and slept with the football coach and she was just horrible…”.
- Difficulties in social relationships (-)
  - “I am scared to meet people because they all seem so strange and their thoughts all seem to be the opposite of mine. I want to breathe a breath that is new [...]. I am going to enjoy this class [psychology] even though is has about a billion people in it and that is a change since my town only has few people living in it...”.
  - “I like all my professors except my freshman seminar prof. He picks on me because I’m quiet and I don’t talk much. I hate when teachers do that. It really bugs me. I like listening to people talk…”.
- Inner dialogues (-)
  - “I never considered myself the type to concentrate on such superficial things. I never considered myself shallow. Maybe I am. Smiles are not shallow, though. Not to me, anyway…”.
  - “I’m just sitting here trying to think of something profound to say. That is stupid considering that I don’t have to say anything important but I guess that is what I’m used to doing when I write an assignment…”.
  - “Why am I being forced to submit my consciousness to the UT psychology department? The entire situation is abhorrent. It is wrong that there is no reward for completing this assignment, it is not part of the grade, it is only taken away from the grade if one does not do it. The current field of the field called psychology frightens and disgusts me of course…”.
  - “[...] Back to the daydream. I enjoy remembering, and have thought about since. I then imagined as though I were past the class [Kendo], but somehow not in the real world as we know it, but somewhere where the sword still ruled the land. I think now it must have been my own idea of feudal Japan, but needlessly I walked around in nothing but a (I forget now what the karate uniforms I have donned so many times are called) but I was wearing one of those, carrying a real sword this time…”.
- Individual activities (-)
  - “I don’t go out partying like most college students. I think I should take advantage of my college years here but I really don’t like the whole clubbing experience. I’m full and I feel like vomiting…”.
  - “Man, I’m hungry. I could go for some fried chicken and biscuits with mashed potatoes and gravy. I wouldn’t mind have some Cajun rice with that. I want to go jogging, but I have too much too read tonight. I should not have registered for morning classes. It sucks having to wake up at eight in the morning and dragging myself to class…”.
  - “This feels kind of good like typing all this. Maybe I’ll start keeping a log or something so I can put all my thoughts in it…”.
  - “I wish I could be doing this in my room so I could be listening to music. The flaming lips which I’m especially into right. I recently bought Zaireeka, a fusion of the words zafire and eureka by them but haven’t been able to play it yet…”.

### Neuroticism

- Negative emotions (+)
  - “I am so depressed. I need a woman…”.
  - “Like today, I feel fat and ugly, so therefore i do not look up when i walk, I don't make any attempt to talk to anyone and when they talk to me I have to pretend to give a shit and be perky, whereas i feel so pressured to say something of meaning or something funny or something that's even remotely interesting that i end up saying less…”.
  - “Don't even know why I m saying this stuff because I'm not suicidal I’m just pissed off and don't have any other way to stop my feelings. I don't know how to stop my feelings…”.
  - “I wish I could be happy in my life. That is all that I ask for and wish for in my life. I feel like nothing really makes me happy. I am not saying I am always sad and my life sucks because this is not the case. I am just saying that sometimes I am sad and maybe sometimes I am content with my life. Content is not good enough for me. I want true happiness and not by myself but with another person. I am trying to find that happiness but it is way out of my reach…”.
  - “I wish you could feel all the hurt that I feel right now. I wish you could hurt so much that you don't know what to do about it. I wish you could take this hurt away from me because I don't deserve to feel this way…”.
  - “Annoyance is about the only word I can think of at this moment to describe how I feel. Today has been awful. Why doesn't anything ever work out the way I wish it would? Everything I needed to accomplish today has been a filure…”.
  - “It is in the afternoon and the only time I have been out of my dorm is too get food. Nothing else so far. That is pretty sad…”.
  - “I'm so scared. I'm sitting here in my own selfpity crying my eyes out and wondering why. my laundry is almost done. [...] why, god? can't I keep him? I miss him. I'm not happy here. I will never be happy again, I fear. not truly happy…”.
  - “But most of all, I really really want to be someone that I want to be that will please everyone and the most of all you. I am so confused. Lord I don't know what to do and this makes me a sad, depressed and unhappy person…”.
- Worries a lot (+)
  - “What should i do with my life, I think my friends don't like me anymore, I am becoming a nuisance to the I think. I tyr my hardest to not mooch off of people, but I always get bad vibes…”.
  - “I hate that hate what else do i hate being lonely, alone or by myself, that's one of my biggest fears being alone and by my self not having a boyfriend or getting married or having a family…”.
  - “Today, bad things done. She would be upset. So many rules broken. What if she surprise visits? Bad. Very bad…”.
  - “I'm overwhelmed with anxiety. I know I'm going to have to get drunk before I go otherwise I'm not going to have anything to talk…”.
  - “I have an eerie feeling that someone I know is loooking over my shoulder, watching me write this stuff that i wouldn’t tell my closest freiends…”.
  - “I want to be able to smile at the world and the world to just smile back. But the world is not that nice. It is such a mean, ugly world that sometimes I wish I didn't live in it…”.
  - “I sometimes wonder if I have what it takes to succeed at ut. My mind is still in doubt seeing the enormous campus each day as I walk to class…”.
  - “They haven't cleaned our carpets and repaired all the messed up parts of the apartment. it's stressful to think about all that needs to be done, and the amount of time left…”.
  - “I’m worried about money, very worried about not following throught and disappointing my parents…”.
- Emotionally unstable, easily upset (+)
  - “How much freaking'longer to I have to sit here this retarded computer better not decide to shut down or there's going to be some shit I'll be pissed ooooooooohhhhhhhhhh I'll be pissed…”.
  - “I hate ballet with a passion and today I felt sick to my stomach in class because I hate it so much. Brandon makes me so mad because he acts gay all the time…”.
  - “I sound like a goddamn theater major being all dramatic…”.
  - “i wish i had more confidence in myself. i have no real reason to lack confidence, i want to be more confident but it seems everytime i am put in a situation where i would need to display confidence i choke. it seems like it is my body's natural reaction to get nervous…”.
- Positive emotions (-)
  - “It seems that me and janet are really going to get along. I hope so. I love her so much; she's so much fun…”.
  - “I love college [...] I love this song [...] I love the wide variety of people you get to see on a daily basis…”.
  - “I have always found it interesting that someone could actually think in another language then they'er own. I used to think when I was a little kid that that would be impossible - then I though itd be incredible, now that I can do taht it just seems like fun, i like it, it feels comforting somehow…”.
  - “That reminds me of a kid who did a debate over the existence of ESP [Extrasensory Perception] in fifth grade gifted/talented class. It makes me feel good to say that I have been identified g/t. That's very cocky sounding, but I don't believe that I have told anyone of my identification in years…”.
  - “The concert last night was great I have never seen anyone play like that especially for that length of time. I mean goodness they played for three and a half hours. Victor is great I would have put incredible but I don't know how to spell it…”.
  - “I hope I start off my college career off with a good semester and then keep it going throughout my college career. That would then lead to many good career opportunities and a happy, successful life…”.
  - “The kind of music where you get a chill running down your spine, and then you realize what a good mood you're in. You can have chill-sending songs that are sad, but'I like the happy ones, they are fewer in number than the sad ones…”.
  - “After i do thins I may go and eat or spend some time with melissa, my girlfriend, who also happens to be my fiance she's great. After that I can write my router advertisement program. That would be fun. I love melissa. Being in austin is great. I like it much better that being in San Antonio…”.
  - “I'm so excited to see him. He's seriously the best thing to happen to me for a really long time…”.
  - “I spent the weekend with my four friends that have an apartment here in austin. My roommate went home for the weekend so I just hung out with them. We had a bbq one night and watched movies and went swimming and laid in the hot tub and just relaxed. It was so much fun. I think that I'll spend this weekend with them again…”.
- Not easily stressed (-)
  - “I don’t know what I would cook them in though. I don’t know if we have a pan I guess Ill find out when I get back upstairs I'm almost done with my laundry. I need to start reading the psychology book too and taking notes. lol, I don’t think I actually think about all the stuff I have to do as much as this writing assignment may make it seem…”.
  - “As these last two weeks have flown by I have realized differnt tecniques to make the rest of my experiences at texas even better. I have learned from my mistakes and I have gained from my experiences…”.
  - “People told me that I would be overwhelmed when I came down here but I haven't felt that way yet. Things are getting harder by the week, but I think I am handling it well…”.

### Openness to Experience

- Artistic, aesthetic experiences (+)
  - “I had my audition for the "madrigal dinner " tonight. I think it went pretty well. I sang a song that I wrote…”.
  - “I am currently majoring in theatre…”.
  - “My history of the banjo teacher, that's a freshman seminar, told us we had to grow our nails out longer to play the banjo, and it's driving me crazy because I hate semi-long nails…”.
  - “Oh Doris Day, music is slowly losing its place or relevance in society; I was raised on 60's and 70's music…”.
  - “I was expecting my writing to be more Ulysses-like; that's a great book: I even brought it to my dorm but haven't had time to read any of it all (not that I did at home, either)...”.
  - “sometimes I hope that my artistic spirit doesn't die the death of a million pinpricks…”.
  - “It definitely reminds of James Joyce, the author of Ulysses who attempted to write an ENTIRE BOOK based on the method of stream of consciousness. Man that was quite painful to read, since he didn't use ANY SORT OF PUNCTUATIONS AT ALL….”.
  - “The song just phased into an ambient sound while Miles is playing over it. The drums, bass, and guitar have dropped out. When the jam comes back in. and here it comes. oh yes. Well done. Movement (change) in music I have realized (as a good explanation) is what creates uniqueness and good sound. Change can include any part of sound. pitch, tempo, tambre, etc. Now it's just the Bass and Miles. On this album is Billy Cobham on drums, Herbie Hancock is on keyboards. I'm not sure who the bass player is off the top of my head…”.
  - “I love art now. There something so mysterious about it to me. What were they thinking? Although I think some of it is sketchy as to call it some sort of meaningful piece but I guess it is to them. It is definitely one of those things left to the beholder…”.
- Active imagination (+)
  - “I wish I had the ability to purr. If only I were a cat…”.
  - “The songs in your head have nowhere to go except leaving the words on the page an I supposed to be speaking in poetry here?...”.
  - “I was thinking about acid the other day, you know, LSD. I was told that Lewis Carroll, author of "Alice in Wonderland", was having one hell of a trip when he wrote the book. That would explain some of the messed up things that he wrote about, but I think that if he had tapped into his own subconscious, he would have been able to write about the same stuff. I mean, LSD allows you to see things that you normally would not see on your own, but I think that deep within your own consciousness, that is what you would really see…”.
  - “I then imagined as though I were past the class, but somehow not in the real world as we know it, but somewhere where the sword still ruled the land. I think now it must have been my own idea of feudal Japan, but needlessly I walked around in nothing but a (I forget now what the karate uniforms I have donned so many times are called) but I was wearing one of those, carrying a real sword this time, trudging through muddy roads through a country side constantly lit by an orange, pasty sun. I can’t remember where it went form there, but needless to say much violence, honor, and success followed. That's how my daydreams sometimes run, but sometime they are more erratic…”.
- Curious about many things (+)
  - “I'm thinking about going into journalism, but who knows? It was the FBI last week…”.
  - “I like foreign languages. rightnow I'm taking dutch- but spanish is the one I'm most versed in. I think in spanish sometimes, its kind of fun, and I have always found it interesting that someone could actually think in another language then they'er own. I used to think when I was a little kid that that would be impossible- then I though itd be incredible, now that I can do taht it just seems like fun, I like it, it feels. Comforting somehow. I'm not sure why. it must be because my goal is to be fluent in as many languages as possible- though I'll probably do only four or five- someone in my class has a father that knows seven. I find hat amazing, it's simply amazing what the mind is capable of…”.
  - “Ellipses are fun, they provide space, and soetimes a depth that no other literary device can reach. I'm not sure if depth is the right word of if tis truly a literary device, but ellipses are (thoughts search for word) something that people use. Woah, a mental stumble in words, I wonder how oftne I do that?...”.
  - “Side note: it's getting hot and I'm turning on the fan. Could this music and the pressure to write as fast as I can be causing my body to heat up? That could be quite interesting…”.
  - “What drives the human race to act the way they do. So ignorant and destructive. As long as I just focus on my life and not that of others, I will be fine. It has got me this far. Life is so wondrous and worth living. Twenty one. the beginning of our end…”.
- Deep thinking (+)
  - “I believe I was talking about time and how controlling it can be when dealt with in the wrong way our whole society is so structured on this intangible object its amazing when you dwell and also can get confusing…”.
  - “It seems to me that everything that an authoritative figure has to say is always an understatement of what is actually expected…”.
  - “I believe that people are too preoccupied with the accumulation of material things. And that the only way to deal with this would be to take away all material things. Like people are not humble and they should be. I'm still in the process of finding ways to implement this idea, but most of these are still on the drawing board. Like if someone created free energy and just gave it out to everyone, I belief that that might completely destroy the economy and that would be good. Money serves no purpose but to bind us to our material things…”.
  - “My typos are me. I make mistakes. I misspell. Rarely of course. I am thinking nothing right now. I wonder if other people do that. Think of nothing. I think of a blank canvas. Is that nothing? I guess not but I believe you can understand what I am trying to say…”.
  - “People laugh at the same things over and over. There doesn't seem to be any diminishing of reward. No matter how long a joke has been around, no matter how old the recipient is; the same things seem to make people laugh. Why is that? Maybe it's because we like hearing the same stories over and over, associating them with our memories. Like "Star Wars". Nobody today looks at Star wars and says, "man, check out those models! No strings? How did they do that?" or, "Man, that Hark Hamill is a great actor!" But most of us can remember a time when we would have said that, so we just watch it over and over and over again. Nobody would do that with "Schindler's List". [...] Come on, "Care Bears" was the most blatantly stupid piece of crap ever to grace the airwaves. Why did we ever like that in the first place? Well, it was the 80's nothing else was on...”.
  - “HOw logical does it sound to make a career descision on a dream? Some might say that it's very logical, like Mom. I love her so much. I remember when I was a freshman in high school and brainwashed by FBBA, and we would get into these huge religious debates. Now I think that there may be more truth to what she thinks than what I believed…”.
  - “Do you ever have the feeling that anger is a mask for pain. I think I play that game a lot…”.
  - “I have also been wanting to put together a compilation of my own philosophies so that I would have a road map to live my life […] Anyway, here I am revealing how my mind works. It is kind of like a release to me. I should do this more often…”.
  - “seems like my consciousness is explaining something to me…”.
  - “Why is it that you can see someone who fits every requirement you have for the perfect mate, but if there's no chemistry there's no relationship at least romantically. Then you might met someone who isn't your type at all, maybe you don't even really like the same music or have the same lifestyle, but if there's chemistry you can't resist each other. Who casts the spells on us? Who laughs at our bumbling confusion? Is it all a matter of having the ideal children? Are the people we have 'chemistry' with the people who have the perfect complementary set of genes to our own? If so, how does my body know? Do my electrons match up perfectly with the spin cycles of theirs?...”.
  - “I don't remember the last time I was free. security. A face is security. can be. Lie down- daydream. sleep things off- give it time, start over. I'm so lonely. need to find someplace in myself- tap into my soul and dig out the pain. You Dwell on answers without thinking and lose yourself temporarily- What is the answer? Hey didn't you know that 42 is the meaning of life, the universe, and everything? Bet on that 42- whatever 42 is. Go with it- run with- and try not to think too hard about the way you see him- the way they curve in your mind- try to fill the empty space beside you with movement- try not to stop and then you won't miss. but that's not true. The Talmud had it right- We do not see things as they are, we see them as we are. And that is the sole truth- even when we think we cannot see ourselves…”.
  - “Sure, some may say that just having played is enough, but for me, to not bring about one's work to full potential is not worth anything…”.
  - “I'm here to tell you that no such place exists, god is just santa clause for adults. Where was god when the twin towers were hit, where was god when the indians were wiped out by the white man, where was god when the germans were killing the jews…”.
- Mundane things (-)
  - “THere is a bunch of paper next to my desk, I wonder who left it. The girl next to me is typing a paper, and another girl just walked in the computer lab. She is wearing a red shirt. I need to eat before the cafeteria closes. It closes really early. The computer next to me keeps turning on and off by itself. Someone else walked into the computer lab. He is wearing a blue shirt and jeans. The screen of the computer to my right is green. The room is starting to feel colder. I need to get my jacket…”.
  - “I left my medicine at home so I'm having trouble breathing. But my mom is sending it to me so I can feel better soon. I have to go to a UT football game for an assignment in my freshman seminar class but I didn't buy a sports package which by the way I think is the stupidest think I've ever heard of. I think if you're a student then you should be able to get into the game for free but what do I know…”.
  - “My favorite subject to study is Calculus because it makes me feel content when I complete a homework assignment and I understood what I did. Especially when I learn and apply a new concept. Even if I didn't understand everything I learned, I go to prof or TA and ask them how to do it. Then I understand the concept and I am satisfied with myself…”.
  - “My ex-boyfriend will also be in town. I haven't seen him in over two months. He goes to West Point in New York and had to leave in late June. I talk to him every once in awhile, but I miss him so much. We are still great friends, and I can't wait to see him again. We dated for a year and three months, so its strange not to see him at all anymore. This gum is really ready to be thrown away, but I'll wait for my four more minutes to do so…”.
  - “that stupid lab stuff is due tomorrow. no maybe it's due Friday I wish I didn't have to take ch 204 it looks like it's going to be the class to give me the most homework I heard it was a weed-out course for Chem E I don't really feel that much stress right now--I should be really scared about not making it as a chemical engineer because my dad, uncle, and sister are all chem e's but I know I can do it and I know the only way I won't make it is if I don't study…”.
  - “I am quite addicted to diet cokes and diet drinks in all, I think I have a serious problem, yet I live for it. I feel like one right now. Boy do I need to do laundry. It just keeps piling up, of course the one day I finally decide to do it, everyone else has the same idea. I can't wait to go through the dorm experience, and then move on into an apartment and have things of my own…”.
  - “I am now looking at my pencil. This pencil has been with me for about a year and I still have not lost it. I usually loose these pens and pencils within 3 or 4 weeks. My roommate just picked up the phone and started talking to a friend of mine. It seems that Texas just won the football game. I am happy to hear that…”.
  - “Being in austin is great. I like it much better that being in san antonio. I want there last weekend and wanted to be back up here the whole time. That could have been because all y stuff is up here. My parents miss me though. I sohuld call them. I;ll do that later. I have a lot of homework to do…”.
  - “The fist thing I have to do before I leave is get all my homework done, including this assignment that I didn't want to leave for tomorrow because then it would keep me awake all night just like all my homework does. Since the first day that I slept in this dorm, it seems that the bed is not for me or something, I cannot fall asleep, and if I do, I wake up every 20 min and look at my clock to see if it's time to go to class…”.
  - “I haven't been to a beach in two summers now because of the knee surgery and rehab (for the knee), and this summer we were just too busy. That reminds me, I miss my family in Virginia. Maybe I can fly out there this summer. That would be fun. Virginia is beautiful. The Chesapeake area especially. I'm glad my brother is alright, but when my mother called it worried me. But he will be fine. I cleaned the guest bathroom tonight (I have to do chores around the house because I live in a co-op)...”.
  - “I need to go exercising I went to the gym yesterday for about 30 minutes and I went walking for about an hour. I am, trying to lose weight for this dorm function on the 27th all these girls are going to be in these skimpy bathing suits and usually wear a T-shirt over mine. Not this time!! I'm gonna wear a bathing suit without a T-shirt what an accomplishment that will be!! I still have to go to the bathroom. Not real bad it's just like this nagging pain in my bladder (sorry that's kinda gross!) But that's exactly what I'm thinking!...”.
  - “I wonder when mom's going to get home. Is she going to the grocery store again? I have to ask Monica about this. I really want some gum. I really wish that mom would get me some. Oh. this cricket is really annoying. Stop making that sound please! Is it already the cricket season again? Last year, I've seen about a hundred crickets, some dead and some alive, in front of Eckerd. Now, we have crickets outside of my bedroom window. Great! Wow. Monica has been watching the TV for a long time now. I'll give her fifteen more minutes and then I'm going to go turn off the TV. She should read more…”.
  - “I will change clothes and go shower or something. I'll get my comb and toothbrush and go to the restroom. What do I wear to go out tonight? I think I will wear my white Polo shirt and my black shorts. No I'll wear my khaki shorts and my white tennis shoes. I can't stay out to long since I have so much homework to do. The entire English rough draft to do, my pre-calculus, and all my psychology reading to do. Man, I feel sleepy, guess I should have gone to bed early last night instead of watching Letterman and Leno…”.
- Don’t enjoy deep thinking (-)
  - “I had fun during critical thinking class. I mean, what is the purpose of taking critical thinking class? I do not get it. However, the class is pretty relaxing--so far. I wonder what the exam is going to be like…”.

## Types

### Introversion - Extraversion

#### Original

##### Introversion (Label 1)

- lose myself completely, starting to rock violently and doing all kinds of awkward movements. After a while, tears often flow from my eyes. I don't know why I cry so much...|||Thanks for writing this. I can relate. Sometimes, in an attempt to connect with people, or in an attempt to describe my inner visions, I create a projection of my true inner self. This may not be...|||This is how the poem relates to me: I am so often swept up in my internal world. Always seeking my passions. I love them more than anything. But sometimes I turn around and realize that there is...|||I get strong scores for 3, 4 and 5 and can relate to all of them. Especially 4w3, 3w4, 4w5 and 5w4. When I first did an enneagram I was amazed on how accurate those four descriptions were (and I...|||Score: 30 10char|||I can relate to everything in your post. I'm also a 24-year old INFP Christian, and I struggle to find out how to reach my dreams despite my introversion. I'm 100 % sure that we can change our...|||First. This post is NOT about supernatural abilities or spiritual gifts. (we could open another thread for that). I remember that someone on this forum wrote that we INFP have the uncanny...|||Wounded Bird I know exactly what you're talking about, I've been in quite a few fi-si-loops myself and I'm deadly afraid of them because they can drain me of all my energy and last for more than 6...|||Let's see... Individualist. Don't follow the crowd. Novel ways of living.

##### Extroversion (Label 0)

- How can you possibly choose?!?!........ I suppose The Rolling Stones, but this is so hard!||| Hell no! I'm the creepy closet lurker :[ *sets up a surveillance camera in your room for when you get back from my...|||You had me at What the fuck are you doing in my closet? <3|||Congrats!! :kitteh:|||Bans you because the customer is always right!|||I've ben here about 8 months now, so I selected 6 months.|||Definitely the mafia sub-forum if that were an option, but overall my favorite section is the entertainment plaza. The Personality Test Resource section is a close second.|||Yay! Congrats MindSlinger !!! :kitteh:|||I lost my teddy bear D:.... Can I cuddle with you instead?|||Well, I think they're probably great. I'm basing that opinion of an 18 year old INTJ friend of mine. We get along great. He's one of the smartest people I know, with the most logical, well thought...|||Haha, yes I was watching the episode, and I saw some of their Facebook posts. It's hilarious how ignorant they are.|||It sounds great! Well, here's hoping someday I actually can live there!|||Well, I like talking to, texting, emailing, and being with people all the time, or even with a group of people all at once. Conversation is usually effortless with people I really like, and even with...|||Yeah, I can completely understand your frustration. My mother and I clash over little things like that all the time and it's really taken its toll on our relationship. The little things can matter a...|||Oh I've heard that before

#### Masked

##### Introversion (Label 1)

- been in any serious situation. ||| alone, away from everyone. can't see anyone, can't hear anyone, can't sense anyone. ||| I wake up early and think for a few hours. If I'm worried about something I'll wake up super early, check the thing I'm worried about, and go back to bed to think. Then I get out of bed, sit on the... ||| I don't dislike them, not really. I don't really talk to them, but I think that's more out of respect for their personal space. ||| Something that could be a part of it is the fact that they are one of the most common types, the most common in females. In today's snow flake obsessed society, it seems that if there are lots of...

##### Extroversion (Label 0)

- you do seem like an [UNK] judging by your posts ||| b: [UNK] m: [UNK] k: [UNK] [UNK], [UNK], [UNK] ||| [UNK] type w8? interesting i am not sure maybe you're an [UNK] ||| k : [UNK] m : [UNK] b : [UNK] [UNK], [UNK], [UNK] ||| k m : [UNK] b : [UNK] [UNK], [UNK], [UNK] ||| it's just a vast difference of interests intelligent people are usually interested in things difficult to understand not because of how difficult they are but because the things difficult to… ||| the guy seems like an [UNK] at first glance so manipulative and a great con who is con at getting people do what he wants to do however i can't help but think [UNK] since he seems to have that [UNK] vibe…||| m b : first [UNK] k : second [UNK] [UNK], [UNK], [UNK] ||| [UNK] hands down v. vtmlt be the center of attention ||| [UNK] (cause they probably don't even know where is everyone going to begin with) tmlt die of heart disease ||| [UNK] tmlt hate thinkers ||| k : [UNK] (bet they'll be good kissers) m : [UNK] b : [UNK] [UNK], [UNK], [UNK] ||| definitely ||| ohhhh *o* forgive my unexcused incompetence i shall work to fix it immediately: laughing: marry: [UNK] kiss: [UNK] (cause like it's gonna be one short relationship before we both move on) bed... ||| [UNK] tmlt turn psycho ||| i would agree to this but [UNK] - [UNK] is hella adaptable in fact it can simulate other functions really good combined with [UNK] - [UNK] i think [UNK] can organize others through playing the devil's advocate i... ||| enxps i guess tmlt impact a great change on the status quo |||

### Intuition - Sensing

#### Original

##### Intuition (Label 1)

- I didnt see where I said anything about jealous- and no Christians dont refer to his as evil- um yeah- what are you 5 years old....|||entjwillruletheworld Since the post was the last was one I didnt feel a need to post as I have in the others- hannibal- said how did intro turn into a war end quote|||I understand what you are going threw. After my divorce I questioned myself alot and still do at times, with many of the questions you asked on here. I felt I had put so much into and made many...|||I dont see a war. I think we are all trying to show the other belief and why|||All stories written in the bible have to be elaborated and made seem more drastic for us to get a clear pic. and a clear insight on what the story holds for us- there is meaning in them all for...|||Peter- Science isnt possible with out the knowledge that was placed with us by God- You seem to have your own explanation for every thing- and I'm glad that you are so certian that science-...|||SKYCLOUDS- Your right god didnt make the the cross for the killing of his son....the non believers did- but do you think its possible that it was already known his child would die and how he would...|||And yes it was made into many religions- but it was taught in the bible that there would be many false religions and the bibe typically speaks of christians

##### Sensing (Label 0)

- always talk to strangers, no matter how creepy they are. attempt to pick pocket as many people as possible. telling your boss off is a good way to blow off some steam. ||| think he was terrible strategically. he could have destroyed the bef at dunkirk but decided to let them evacuate. then hitler decides to shift the battle of britain to bombing london hoping they... ||| spaghetti and garlic bread. ||| welcome to the forum. ||| welcome to the forum. ||| welcome to perc. it took me a couple tries to get a better understanding of which type i am. ||| exactly. a good looking person for example could still be insecure. ||| physically or mentally ? ||| isfj and i add two spoons of sugar and two of cream. ||| cremated and to have the ashes thrown into a waterfall to symbolize how life is a series of events that moves from one moment to the next. ||| marinated steak, mashed potatoes, corn on the cob and home made apple crisp for dessert. ||| welcome to perc. ||| welcome to perc. ||| welcome to perc. ||| welcome to perc. ||| welcome to perc. ||| welcome to perc. ||| welcome to perc. ||| welcome to the forum. ||| welcome to the forum. ||| welcome to the forum. ||| welcome to the forum. ||| understand that worry is just made up thoughts in your head that you created. ||| my dad's good one liner you always need to take care

#### Masked

##### Intuition (Label 1)

- Well, Germany is absolutely and unquestionably a social culture, though the prussian character is fixed. Switzerland is the German-speaking country, Austria is. About Germany there is... ||| yes, I found out from a mutual acquaintance that this is true - - bliss stream owns stack me up. It brings up a significant issue, in that I had found his sp/sxw1 description to describe someone else I... ||| I decided to actually read what Beatrice chestnut has to say about the place of shame in the image (sorry, sadness) triad. I'm finding chestnut's book to be a lot more valuable when she just focuses... ||| for instance, there is this in her chapter on sixes about social sixes: they tend to be shy and have little ability to socialize or to be moved or touched by something or someone. Really? this... ||| now that's... interesting. I mean... seriously... where do you guys think the envy, deceit, and pride come from? What do you think they're for? What do they compensate for? What do they cover up?... ||| hopefully she will explain that in denying the primacy of shame in the image triad, she is also denying its actually being the image triad. Because the shame comes from knowing - - consciously or... ||| I want to thank everyone who's participated on or followed this thread. It acquired a depth of meaning which I never could have anticipated. This began for me with the discussion of Florence welch... ||| the trimrti (english: three forms; sanskrit:trimrti), trimurati or trimurati, is a concept in hinduism in which the cosmic functions of creation, maintenance…

##### Sensing (Label 0)

- this is not like me at all. just sayin'... ||| hello sub - forumites, i haven't logged on in a while. work has kept me busy among other things like instagram and twitter (my two new go - tos). this thread is one of the top on the list of threads so... ||| pictures ? hmm.... i kinda like the anonymity. i'll think about it. i'm not that photogenic. i'm just a dude. ||| imo.... douchebag alert ! douchebag alert ! seriously ? find someone who is a gentleman. this guy is immature and is just playing around with people's feelings. i say stick him in the friend zone... ||| on the other hand, i don't like starting conversations with strangers. lately, i've noticed strangers talking to me first. though i default as an extrovert, i don't find it necessary to talk to... ||| i'm sure this goes beyond personality and there's most likely more to her story as to why she bahaves (ed) that way. anyone can have an angry disposition regardless of their personality. ||| zombie, you're one of my faves on perc. see, you're an [UNK] and i don't dislike you. for the most part, [UNK] are peacemakers so we will default to the most practical and efficient way of... ||| i'm really bummed my post looks like that. and when i tried to edit it, it wouldn't save it. i gave up. sorry hunny. ||| thanks for the invite hunnybunny. & nbsp ; i've read all the post and feel caught up between you and zombie and thequirkygirl (who is ironically a dude who is former military). <br> much like zombie, i do... ||| i'm back and here are my results. first, i just

### Feeling - Thinking

#### Original

##### Feeling (Label 1)

- Paragon: I guess it's always a possibility, but I am certain I am INFP. I'm very close to 50/50 E/I, but I'm on the Introverted side. I considered ENFP for a long time, but it didn't sit right. My...|||I have a really hard time expressing myself effectively, especially when I feel something very deeply. It's like it's stuck there...left to fester and dig deeper until I feel sick with it (which I...|||@ Rather Unusual Yikes! I'm sorry you feel this way. :( I've never realized guys felt inferior like that; and now that I recognize it, I know you can't be alone. I can see where you are coming...|||Faeriegal has a great post (and personal experience!)..but I thought I'd share my thoughts as well. I used to consider nursing...and I'm in the same boat as you are with trying to decide my life...|||I am curious why you think Drizzt would be INFJ. He has a very strong Fi (rather than Fe) function in my opinion. I've always thought him to be INFP/ISFP. What makes him INFJ? :)|||[>.<Sorry, dunno how to delete this.]|||I am obsessed with the concept of being as in tune with your natural and true self as possible. The ideals of society aggravate me; how people can be so blind...and shape themselves to society's...|||I read that Captain Ahab in Moby Dick is INFP. :)

##### Thinking (Label 0)

- if you do the person will probably just blow up in your face....|||So if you consider a man an animal and humans are animals, what does that make you? You're not an alien are you? :crazy: Yes, you could say the same thing with sex. There are consequences to it...|||Yes, I am subconsciously attracted to good hips and thighs. The measurement is somewhere along the lines of this girl, she has fantastic hips and thighs. The hips and thighs theory is not BS, I am...|||Yes, I like high standards but it's just not practical enough. If I'm attracted to a woman then I just am. I didn't selectively take the time to think about it and choose to be. My brain fired off my...|||Yes, probably someone purposely told others that it's a brain test and people believed it. Probably a bad ENTJ told someone that it was a brain test, okay which one of you ENTJ's is responsible for...|||People actually misinterpret the spinning lady as a left brain/right brain form of test. It is actually created as an optical illusion and often mistaken as a test.|||Self-deprecation? No. But when I do feel the need for more information I ask intriguing and challenging questions to arouse the interest of the rest of the people around me. As far as prefacing a...|||You mean Mormons?|||Peregrine Falcon.|||Because when you become an English teacher you become the master of English grammar? Then you can smite and crush the stu...errr...I mean then you can teach English and grammar properly to the...|||

#### Masked

##### Feeling (Label 1)

- are many advisers within me: ratio/logic, psyche and emotional needs, morals, expectations of other/close people, bodily needs/demands, worldview and mindsets, gut feelings, intuition,... ||| I believe [UNK] with well developed [UNK]... - have left behind the victim identity - are not only sincere and authentic but also competent - Don't only desire to make a change in the world, but... ||| I hate when I have a fight with my imaginary wife and then feel down all day... ||| I'm horrified at how quickly I can lose the connection to my heart, to the real core of who I am and what truly matters. There are so many ways to spend my time and C can get so caught up in the...

##### Thinking (Label 0)

- if you do the person will probably just blow up in your face.... ||| so if you consider a man an animal and humans are animals, what does that make you ? you're not an alien are you ? : crazy : yes, you could say the same thing with sex. there are consequences to it... ||| yes, i am subconsciously attracted to good hips and thighs. the measurement is somewhere along the lines of this girl, she has fantastic hips and thighs. the hips and thighs theory is not bs, i am... ||| yes, i like high standards but it's just not practical enough. if i'm attracted to a woman then i just am. i didn't selectively take the time to think about it and choose to be. my brain fired off my... ||| yes, probably someone purposely told others that it's a brain test and people believed it. probably a bad entj told someone that it was a brain test, okay which one of you entj's is responsible for... ||| people actually misinterpret the spinning lady as a left brain / right brain form of test. it is actually created as an optical illusion and often mistaken as a test. ||| self - deprecation ? no. but when i do feel the need for more information i ask intriguing and challenging questions to arouse the interest of the rest of the people around me. as far as prefacing a... ||| you mean mormons ? ||| peregrine falcon. ||| because when you become an english teacher you become the master of english grammar ? then you can smite and crush the stu... errr... i mean then you can teach english and grammar properly to the... ||| true, there

### Judging - Perception

#### Original

##### Judging (Label 1)

- ESTJ's get all the credit, but ESFJ's do the same thing WHILE managing the emotional...|||Interestingly, we did have some fights where I finally blew my lid, and I acted in a way that I was HORRIFIED by after the fact (honestly, even as I was doing it, but I felt completely out of control...|||I can second this experience (INTJ male living with ESFJ female). We had some conflict for a while after we moved in together simply because her tone was received (by me) as being much more...|||The key to any type re-finding their center and regaining balance is the auxiliary function - in this case for an INTJ, that Extraverted Thinking. He needs to (at some point) stop dwelling on how he...|||I'm INTJ in a relationship with an ESFJ. The articles you linked to are fantastic. My experience is that when an ESFJ gets sufficiently mad, they just stop thinking right. I'm not trying to be...|||Emma, I'll share what I can from my own experience and personal analysis. I'm an INTJ male, I'm dating (really, living with) an ESTJ female. You're right, on the surface it is a TOTALLY wacked out...|||As an INTJ who has been intimately acquainted with both types, I can tell you - No. Not at all. They aren't even sort of close. Just... no.|||I think the dynamic of the stereo type is that INTJ's have a preference for depth, with a tendency to not act enough. ESTJ's have a preference for decisive action, with a tendency towards...

##### Perceiving (Label 0)

- overactive left brain hemisphere and underactive right brain hemisphere. Feeling is an overactive right brain hemisphere and...|||Haha, I agree. PS: Do you know how much your avatar looks like Trevor Philips from GTA V?|||Cmon, who butters their toasts, honestly... Also, I don't mean to question your judgement or anything, but are you sure he's not ENFJ? I can't really see ENFP's being fluffy since our F is...|||I knew she was either INFJ or ISFJ (introverted Fe users). My best friend was an INFJ too, I've found out that the ENFP is the doormat in these relationships. I don't know why, but when you frickle...|||I'm finding Johnny Depp the most obvious famous Ne user. But I'm pretty sure he's more ENTP than ENFP.|||@MuChApArAdOx Yes, I also view ENFP women as more alpha than most other types, but only because you're generally the more feminine women (which goes hand in hand with the (E)NF temperament), not...|||@The_Wanderer I don't care about sports either (atleast following/watching them, playing them I like!). I generally thought that this was our N coming in the picture. I can't imagine an N-dominant...|||No, you're just another one of them immature ENTP's who are extremely good at overestimating their potential. ENTP's are never world dominators simply because of your P. Leadership is out of the...|||Because you're a pushover. Bullies only bully pushovers. You can stop being a pushover by building self-esteem. Working out/exercise does wonders for your self-esteem.|||That's simple. Just ask him directly on a date. Not speaking for all ENFP males here but.

#### Masked

##### Judging (Label 1)

- Understand why I am this way ^ ||| myself - my ex - one of my nephews and one of my nieces are [UNK]. The only time anyone would ever have the opportunity to meet one of us would be at our home, work, or school. We do not go to social... ||| I hate being expected to hug and I hate being hugged. Only close family members get hugs or are allowed to hug me. Even at that, I still feel like gggrrr. ||| Sometimes. ||| I remember especially social security numbers. haha! ||| very true. Never easily but deeply yes. ||| [UNK] are fun to be around. I greatly enjoy their company. In fact, I really really like an [UNK] but he mentioned once that the feeling was not mutual. I left it as is. Not to say, that I gave up or... ||| Why, of course! you know he love you. Since we love the person, we don't mind. We just laugh at ourselves inside as well. ||| I totally agree with the snapping. I have been called out on this numerous times at work especially. It is just part of me. The death stare as well. ||| [UNK] usually don't keep close connection with their ex's. I don't. It took me almost five years to change my mindset about my [UNK] ex. He doesn't use harsh words but he takes action in cold and... ||| Door slamming is possible with all circumstances. ||| not really. ||| wake up. Turn on my computer. Go onto youtube and put on my playlist. Take a shower. Breakfast. Read read read. Log into my facebook. Write my thoughts in my notes that I keep

##### Perceiving (Label 0)

- crook who is about as smart as a box of rocks and police who aren't much smarter.... a few... ||| i used to feel like a zombie, now i just find ways to scare the shit out of myself regularly. usually snowboarding or rock climbing. a lot of times just getting out and fishing or even offroading... ||| my left ear is completely clogged. not being able to hear out of one ear is the most annoying thing ever. ||| i once had one night stands in a row. the first two were great. i just got up in the morning, said bye, and walked home. the third was pretty uncomfortable because i woke up alone on the couch... ||| i also think people envy our rationality and mystery. when we feel the need to say something important, people listen. i've noticed too many times people tune out really extroverted types of... ||| sometimes i really need to get away. my favorite thing is to drive up to the mountains and fly fish trout in very remote areas. i actually prefer to fish alone because fly fishing is like a puzzle... ||| i used to be pretty wild, like months ago. then i got arrested, totaled my truck, and got a shit load of misdemeanor driving tickets. license suspension, fines, tow bills, and multi - year probation... ||| damn i live in a beautiful state. a couple of my pictures. don't have any of my pics of the tetons on my computer, but grand teton national park might be my favorite place ever.... ||| attractive, relatively low maintenance, adventurous, of at least average
